# Stability study of organometal halide perovskite and its enhanced X-ray scintillation from the incorporation of anodic TiO_2_ nanotubes†

**DOI:** 10.1039/d0ra08881a

**Published:** 2020-12-08

**Authors:** Hui Li, Zhenhua Chen, Zhuocheng Sang, Xiangzhi Zhang, Yong Wang

**Affiliations:** Department of Optoelectronic Science and Engineering, College of Science, Donghua University Songjiang District Shanghai 201620 China; Shanghai Synchrotron Radiation Facility (SSRF), Shanghai Advanced Research Institute, Chinese Academy of Sciences Shanghai 201800 China chenzhenhua@zjlab.org.cn

## Abstract

Organometal halide perovskite-based optoelectronic devices are currently a hot research area owing to their unique properties, but widespread commercialization is plagued by their poor long-term stability. So far, the degradation mechanism of organometal halide perovskites is still indistinct due to limited real time systematic study. In this work, we *in situ* study the crystal evolution of an organometal halide perovskite CH_3_NH_3_PbI_3_, which is prepared on different kinds of framework substrates. Based on the *in situ* grazing incidence X-ray diffraction and X-ray near absorption edge spectrum, we observe the formation of some 2D networks of [PbI_6_]^4−^ octahedra intermediates during CH_3_NH_3_PbI_3_ degradation in a moist environment at the early step of the degradation mechanism. We also show that the structural stability of CH_3_NH_3_PbI_3_ deposited anodic TiO_2_ nanotube substrates is relatively better than that of prepared perovskite on TiO_2_ nanoparticles in moisture. The confinement of the 3D [PbI_6_]^4−^ octahedral crystal network probability reduces the ion migration by regular pores of crystalline TiO_2_ nanotubes, improving the stability of the organometal halide perovskite. Furthermore, the X-ray excited luminescence intensity of CH_3_NH_3_PbI_3_ fabricated on TiO_2_ nanotubes is boosted 88% compared with that of conventional TiO_2_ nanoparticle substrates, which demonstrates its potential application in scintillation detectors.

## Abstract 2

Organometal halide perovskite-based optoelectronic devices are currently a hot research area owing to their unique properties, but widespread commercialization is plagued by their poor long-term stability.

## Graphical abstract

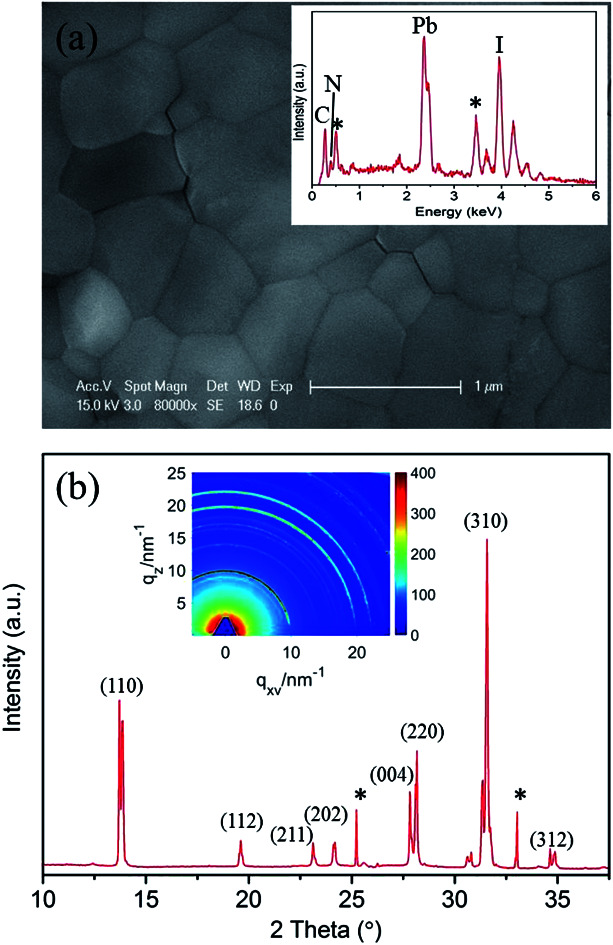

## 1. Introduction

Organometal halide perovskites, CH_3_NH_3_PbX_3_ (X = Cl, Br, I), have become a popular topic with regard to optoelectronic devices in recent years. These perovskite structured semiconductors have benefitted from unique properties, including suitable bandgaps,^1,2^ high absorption coefficients,^3^ long charge carrier diffusion lengths,^4^ and high carrier mobility with low recombination rate.^5–7^ The perovskites thus attract progressive attention for potential applications in fields such as solar cells,^8–14^ wavelength tunable light emitting diodes (LEDs),^15–21^ microlasers,^22,23^ X-ray photodetectors and scintillation.^24–30^

Despite significant progress in organometal halide perovskite-based optoelectronic devices, widespread commercialization is plagued by their poor long-term stability. Because of the soft nature of organometal halide perovskites, perovskite devices are very sensitive to environmental factors such as light, heat and water vapor, which greatly reduce their working stability.^12,31,32^ Among these issues, moisture is one of the most important factors that rapidly degrades organometal halide perovskites, resulting in a significant decline in device performance.^33^ Yang *et al.* reported the photo conversion efficiency (PCE) dropped 80% over a 24 h period when the cells were stored under ambient conditions and a 95% drop in PCE after 6 days for unencapsulated perovskite solar cells.^8^ Seok *et al.* reported that CH_3_NH_3_PbI_3_ began to decompose under the condition of 50% humidity, and its color gradually changed from dark brown to yellow, accompanied by the degradation of the device efficiency.^34^ In organometal halide perovskite-based light emitting diodes (LEDs), the degradation of perovskites induces ion movement, resulting in structural distortion and luminescence wavelength shift, and the peak efficiency of perovskite LEDs is only seconds or minutes under operation.^35^

In the degradation study of organometal halide-based optoelectronics, Wang *et al.* reported that the combined action of water and oxygen led to irreversible decomposition of perovskites. Perovskite CH_3_NH_3_PbI_3_ is extremely sensitive to moisture, easily producing CH_3_NH_2_, HI, and PbI_2_ by hydrolyzation. The presence of oxygen further oxidizes HI to I_2_, resulting in rapid decomposition of perovskite.^36^ Yang *et al.* demonstrated the formation of a hydrated intermediate compound (CH_3_NH_3_)_4_PbI_6_·2H_2_O in the degradation process using grazing incident X-ray diffraction (GIXRD), followed by irreversible decomposition to PbI_2_.^37,38^ In addition, Walsh *et al.* believes that water molecules first combine with CH_3_NH_3_PbI_3_ and extract protons from ammonia to generate an intermediate substance [(CH_3_NH_3_^+^)_*n*−1_(CH_3_NH_2_)PbI_3_]·[H_3_O]. The intermediate product is decomposed into HI hydrate and volatile methylamine, which cause the degradation of perovskite and eventually generate PbI_2_.^39^ At present, the degradation of lead halide perovskite in a moist environment is still inconclusive, especially because the evidence of intermediate substances is not sufficient at the start of moisture exposure. The *in situ* study of the degradation process with multiple methods is critical to understand the degradation process and promote ways to enhance perovskite stability.

In this work, we study the degradation of the organometal halide perovskite CH_3_NH_3_PbI_3_ on both conventional porous TiO_2_ nanoparticles (NPs) and anodic TiO_2_ nanotubes (NTs) to locate the intermediate substance and evaluate the substrate effect on degradation in a moist environment. Both the surface and bulk crystal structure evolution of such perovskites is investigated using synchrotron-based high-resolution 2D GIXRD with different incident X-ray angles in controlled moisture *in situ*. First, the organometal halide perovskite CH_3_NH_3_PbI_3_ was prepared on conventional TiO_2_ NPs. Both the surface and bulk crystal structure of the perovskite film were studied using *in situ* 2D GIXRD under controlled humidity. The intermediate structure was observed once the CH_3_NH_3_PbI_3_/TiO_2_ NPs were exposed within a relative humidity of 50%. Combined with the *in situ* GIXRD and X-ray absorption near edge structure (XANES), the intermediate structure was determined to be a 2D network of [PbI_6_]^4−^ octahedra. Next, the anodic TiO_2_ nanotube arrays served as substrates to prepare CH_3_NH_3_PbI_3_ films, and the *in situ* GIXRD conducted in controlled moisture confirmed that the intermediate structure of CH_3_NH_3_PbI_3_/TiO_2_ NTs appeared at a higher humidity than the CH_3_NH_3_PbI_3_/TiO_2_ NPs. The regular pores of TiO_2_ nanotube arrays were confined to the [PbI_6_]^4−^ octahedral, reducing ion migration and octahedral agglomeration. The stability and crystallinity of the assisted organometal halide perovskite were improved correspondingly. Finally, the scintillation properties of the CH_3_NH_3_PbI_3_/TiO_2_ NPs and CH_3_NH_3_PbI_3_/TiO_2_ NTs were investigated by the X-ray excited luminescence (XEOL) spectrum using 40 keV incident photon energy. The peak luminescence of CH_3_NH_3_PbI_3_/TiO_2_ NTs was improved 88% compared with the conventional TiO_2_ nanoparticle substrate.

## 2. Experimental section

### 2.1 Growth of anodic TiO_2_ nanotubes

The titanium (Ti) sheets (0.125 mm thick, 99.9% purity, Alfa) were ultrasonically cleaned in acetone, ethanol and deionized water for 10 minutes, respectively. And then the sheets were dried in air before anodization. The electrolyte during the anodization was 0.27 wt% NH_4_F dissolved in a mixed solution of ethylene glycol (≥99.5% purity, Riedel-de Haen) and deionized water with volume ratio of 50 : 1. The electrolyte was aged for 12 hours before use. The anodized TiO_2_ nanotubes were made in a two-electrode electrochemical cell, which defined a working area of 1.5 cm^2^. The Ti sheets were employed as the anode and the Pt gauze was taken as the counter electrode. The anodization was performed for 1 hour with a constant voltage of 60 V powered by Keithley 2450 Sourcemeter. After anodization, the TiO_2_ nanotubes was rinsed with ethanol for several minutes, and then dried in air. Finally, the as prepared TiO_2_ nanotubes on titanium sheets were annealed in air for 2 hours, so that the amorphous TiO_2_ nanotubes were transferred to crystalline structure.

### 2.2 Preparation of perovskite CH_3_NH_3_PbI_3_ film

Hydroiodic acid (57 wt% in water) and methylamine (40 wt% in methanol) with 1 : 1 volume ratio were stirred in an ice bath for 2 hours. The solution was evaporated at 50 °C for 1 hour to obtained CH_3_NH_3_I precipitate, which was washed three times with diethyl ether and then dried under vacuum. To prepare (CH_3_NH_3_)PbI_3_, CH_3_NH_3_I and PbI_2_ (99.9%, Sigma-Aldrich) with mole ratio 1 : 3 were mixed in dimethylformamide (DMF) solvents (99.9%, Sigma-Aldrich) at 75 °C with stirring for more than 6 hours in glove box.

All films were fabricated on precleaned FTO glass substrates, convention TiO_2_ nanoparticles film, or Ti sheets assisted TiO_2_ nanotubes with one step spin-coating method. The 2 mL CH_3_NH_3_PbI_3_ perovskite dissolved DMF solution was dropped onto the substrates. A standstill for 30 s is adopted to infiltrate the perovskite solution into substrates, followed by a spin coating with 3000 rpm for 40 s to generate a 300 nm film. The spin-coated perovskite films turned to black after being dried on a hot plate at 70 °C for 30 minutes in a glove box.

### 2.3 Synchrotron based *in situ* GIXRD and XANES study

*In situ* GIXRD experiments were conducted at the X-ray diffraction beamline (BL14B) at the Shanghai Synchrotron Radiation Facility (SSRF). An energy of 10 keV (*λ* = 0.124 nm) was selected using a Si(111) monochromator. A 2D MAR CCD detector was mounted on the six circle Huber diffractometer arm placed at a distance of 268 mm from the sample. A lead beamstop was used to block the direct beam. For *in situ* experiments, a custom made humidity controlled sample chamber was placed on the diffractometer (ESI Fig. S1†). Wet nitrogen through an aqueous solution contributes the moisture environment, and valves are used to control the proportion between dry and humid nitrogen. The measurement accuracy is upto 0.1% by a high accurate humidity sensor. The perovskite films were placed in the chamber sealed with a Kapton window with controlled humidity, and the microstructure evolution of the film can be observed *in situ* under a set humidity environment. To minimize the effect of air on the film before the *in situ* GIXRD test, the sample was placed in a glove box and then transferred to the synchrotron X-ray diffractometer for experiments by a sealed chamber.

Soft X-ray based XANES for the nitrogen K-edge study was carried out on the soft X-ray scanning transmission X-ray microscopy beamline (BL08U) at the SSRF. The XANES was recorded using a total electron yield (TEY) mode, and the photon flux of the incident beam (*I*_0_) was monitored simultaneously based on the photocurrent of the Au grid located before the entrance of the end station under vacuum conditions of 1 × 10^−5^ torr. The X-ray Absorption Fine Structure (XAFS) study of Pd L-edge was carried out on BL14W at the SSRF using a fluorescence mode.

### 2.4 Characterization and X-ray scintillation study

Morphologies of the TiO_2_ nanoparticles, TiO_2_ nanotubes, and CH_3_NH_3_PbI_3_ perovskite films were observed by a field emission scanning electron (SEM) microscope. X-ray photoelectron spectroscopy (XPS) data of the perovskite sample was obtained by an Escalab 250Xi (Thermo Scientific) spectrometer with an excitation source of Al-Kα radiation. X-ray scintillation study was carried out at the X-ray Imaging beamline (BL13W) at SSRF. A 40 keV monochromatic X-ray beam with spot size of 500 μm × 500 μm was used to illuminate the organometal halide perovskites, CH_3_NH_3_PbI_3_ and CH_3_NH_3_PbBr_3_, while the light emitted was collected by an optical fiber of diameter 600 μm and transferred to a spectrometer (HRS-300 MM, Princeton Instrument, USA) with a slit of width 200 μm. The spectrometer housed two gratings with line density of 300 G mm^−1^ and 1200 G mm^−1^, covering the visible and near infrared light wavelength from 200 to 1000 nm.

## 3. Results and discussion

**Fig. 1:**
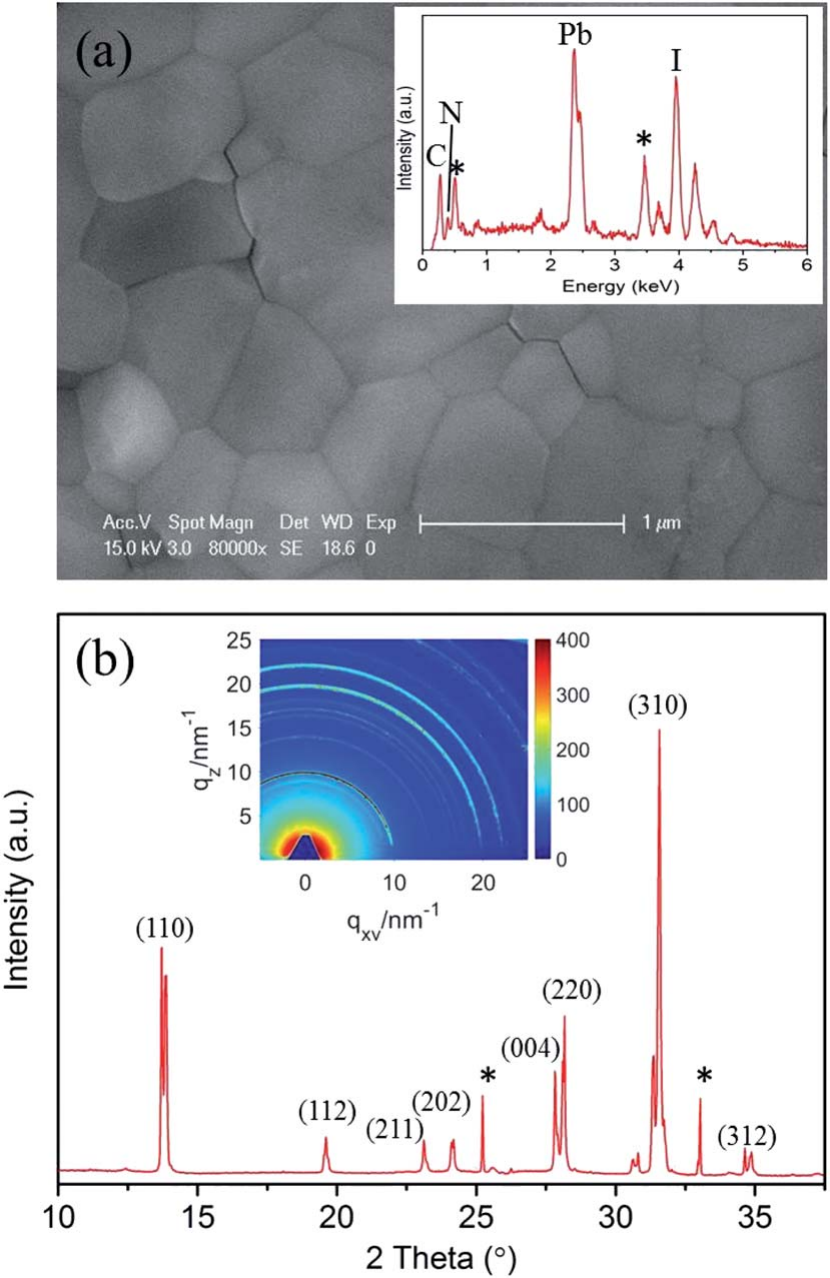
(a) SEM image of the as-prepared CH_3_NH_3_PbI_3_ film on FTO glass. The inset is the EDX spectrum of the CH_3_NH_3_PbI_3_ film. (b) XRD spectrum of the CH_3_NH_3_PbI_3_ film extracted from the 2D GIXRD pattern in the out-of-plane direction. The inset is the 2D GIXRD pattern in *q*-space taken with an incident X-ray angle of 0.2°.

CH_3_NH_3_PbI_3_ perovskites were prepared by reacting methyl ammonium iodine (CH_3_NH_3_I) and lead iodide (PbI_2_) in DMF solution through a one-step method.^3^ The morphology of the prepared CH_3_NH_3_PbI_3_ film is shown in Fig. 1a. The surface of the film is smooth and flat. Energy-dispersive X-ray spectroscopy (EDX) analysis (inset of Fig. 1a) demonstrates that the prepared perovskite sample has an elemental composition of C, N, Pb and I. Herein, the EDX spectrum labeled by stars indicate the O (0.52 keV) and Sn (3.44 keV) elements that stem from the bottom substrate of fluorine-doped tin oxide (FTO) glass. The elemental composition was further confirmed by XPS measurements, the analysis suggests a joint contribution of Pb, I, N and C in prepared CH_3_NH_3_PbI_3_ film, whereas O is presented in low amounts, see ESI Fig. S2.†

The corresponding X-ray diffraction (XRD) spectrum of the CH_3_NH_3_PbI_3_ film is shown in Fig. 1b. The spectrum was extracted from the 2D GIXRD pattern in *q*-space taken with an incident X-ray angle of 0.2° (the inset of Fig. 1b), and the peak positions match well with those of the reported tetragonal CH_3_NH_3_PbI_3_ perovskite structure and the calculated *a* = 8.855 Å and *c* = 12.659 Å.^40,41^

**Fig. 2:**
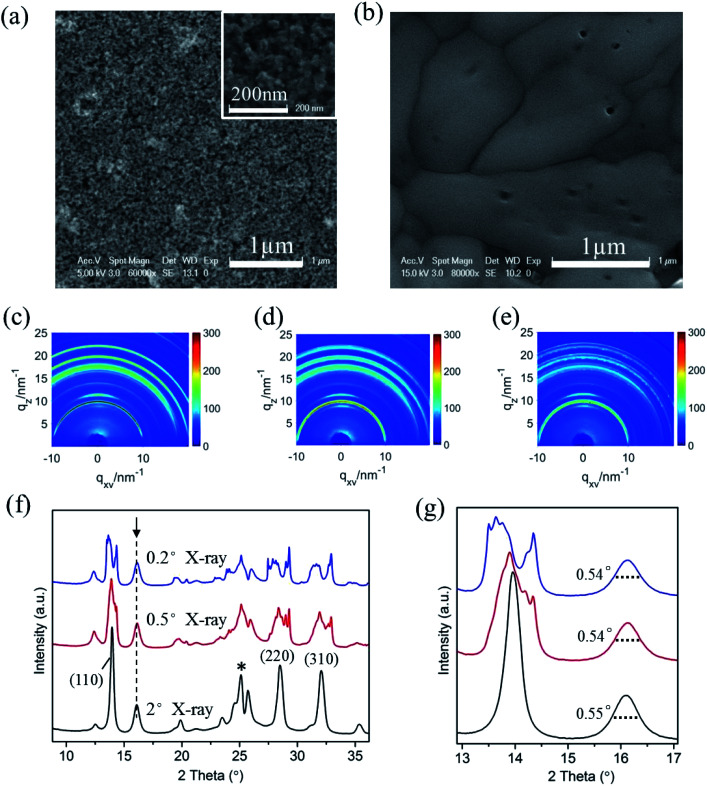
SEM images of (a) bare TiO_2_ nanoparticle film and (b) fabricated CH_3_NH_3_PbI_3_ films; the inset of (a) is a high magnification image of porous TiO_2_ nanoparticles. (c)–(e) The two-dimensional diffraction signals of the CH_3_NH_3_PbI_3_/TiO_2_ NP films exposed to 55% relative humidity using incident measurement grazing angles of 2°, 0.5°, and 0.2°. (f) The dimensional diffraction spectrum integrated from (c–e) in the out-of-plane direction. (g) The magnified XRD spectrum of (f) for obtaining the FWHM of the crystal planes.

In optoelectronic device applications, CH_3_NH_3_PbI_3_ films are usually grown on a titanium dioxide frame. Therefore, to investigate the film structure evolution under the humid atmosphere by *in situ* GIXRD measurements, CH_3_NH_3_PbI_3_ films were prepared on conventional TiO_2_ nanoparticle films in the first step. The SEM images of the bare TiO_2_ nanoparticle film and following fabricated CH_3_NH_3_PbI_3_ films are shown in Fig. 2a and b, which confirm the uniformity of the prepared CH_3_NH_3_PbI_3_ films.

We used different incident X-ray grazing angles to study the evolution of the crystal structure from bulk to surface in moisture environment. Fig. 2c–e shows the *in situ* GIXRD pattern of the perovskite film in an environment of 50% relative humidity with grazing angles of 2°, 0.5° and 0.2°. The three main peaks at the diffraction vector *q* = 10, 20, 22.1 nm^−1^ correspond to the (110), (220), and (310) planes of the tetragonal perovskite crystal structure.^42^Fig. 2f is the one-dimensional diffraction spectrum corresponding to Fig. 2c–e, obtained by azimuth integration in the out-of-plane direction. An additional peak at 16.1° (*q* = 11 nm^−1^) can be observed once the CH_3_NH_3_PbI_3_/TiO_2_ NPs are exposed to 50% relative humidity, irrespective of the X-ray incident angle. This peak indicates a new intermediate structure that is different from the reported PbI_2_ and CH_3_NH_3_I.^43^ The uniform diffraction ring with high intensity along the out-of-plane direction in Fig. 2c–e confirms that the intermediate has anisotropic planes parallel to the substrate.

CH_3_NH_3_PbI_3_ typically has a tetragonal perovskite structure (space group *I*_4_/*mcm*) at room temperature.^37,44^ The methyl ammonium CH_3_NH_3_^+^ cations are localized to the area between the two [PbI_6_]^4−^ octahedral layers. The organic and inorganic components interact by hydrogen bonds between the amino group and the halide ions, whereas weak van der Waals interactions exist among the organic components. Depending on the occupation and orientation of the organic amino cations, the spacing between the inorganic layers is accordingly changeable. When the organometal halide perovskite CH_3_NH_3_PbI_3_ is exposed to a humid environment, the adsorbed water molecules on the perovskite surfaces rapidly extract protons from organic amino cations. Due to the soft nature of organic CH_3_NH_3_^+^ cations, intrinsic degradation occurs in perovskite materials upon the breaking of the bonds and with the generated excitation stresses, producing some 2D [PbI_6_]^4−^ octahedral pieces from the partly cracked 3D [PbI_6_]^4−^ octahedral network. The newly denoted XRD peak at 16.1° (*q* = 11 nm^−1^) matches the reported 2D [PbI_6_]^4−^ octahedral crystals.^45,46^ This finding is basically in accordance with the report by Kelly *et al.* that the distortion of [PbI_6_]^4−^ octahedrons leads to a multiphase structure in perovskite crystal degradation.^37^

In addition, it is observed that the (110), (220), and (310) planes are split obviously for the CH_3_NH_3_PbI_3_/TiO_2_ NPs when the X-ray incident grazing angle decreases from 2° to 0.2° (Fig. 2f) in 50% RH moisture. This is attributed to the large and wide distributed crystal grains of perovskite in the direction perpendicular to these diffraction surfaces. In general, sharp diffraction peaks originate from the large grains, which lead to discontinuous 2D diffraction rings and separated peaks at small grazing angles. Besides, the stress located at the large perovskite CH_3_NH_3_PbI_3_ crystal surfaces may also results in their planes split at low incident grazing angle, especially the prolonged spot size enlarged surface stress effect.

The crystallite sizes of the intermediate 2D [PbI_6_]^4−^ intermediates can be obtained quantitatively from their respective enlarged XRD patterns (Fig. 2g) by using the Scherrer equation:^47,48^
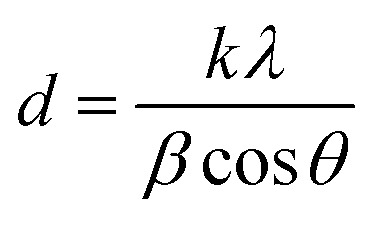
where *d* is the crystallite size in nanometers; *k* is the shape factor constant, which is 0.89; *β* is the full width at half maximum in radians; *λ* is the wavelength of the X-rays, which is 0.124 nm for the incident synchrotron X-rays; and *θ* is the Bragg diffraction angle. The intermediate crystallites are calculated to be ∼11.6 nm in such CH_3_NH_3_PbI_3_/TiO_2_ NPs. The peak of the intermediate 2D [PbI_6_]^4−^ octahedral crystals is not split at even small X-ray grazing angle of 0.2°, which confirm the size distribution of the intermediate crystallites is relatively uniform.

Furthermore, the characteristic peak intensity of CH_3_NH_3_PbI_3_ is decreased in the small X-ray grazing angle (0.2°) GIXRD measurement, while the peaks of nondegraded perovskite samples are not changed obviously (ESI Fig. S3†). This result provides evidence that the CH_3_NH_3_PbI_3_ perovskite crystals are degraded from the surface to the bulk, which indicates that the induced lattice stress released privilege from the high-energy perovskite crystal surfaces.

**Fig. 3:**
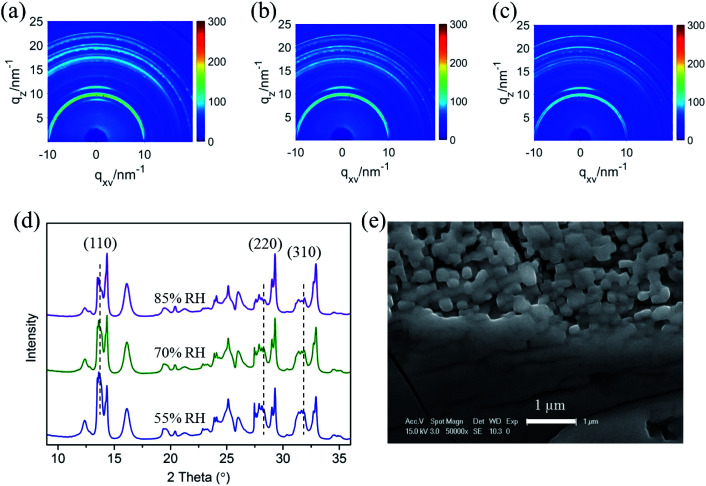
(a)–(c) The two-dimensional diffraction signals of the CH_3_NH_3_PbI_3_/TiO_2_ NP films exposed to 55%, 70%, and 85% relative humidity using an incident measurement grazing angle of 0.2°. (d) The one-dimensional diffraction spectrum integrated from (a–c) in the out-of-plane direction. (e) SEM image of CH_3_NH_3_PbI_3_/TiO_2_ NP film after the *in situ* crystal study in a moist environment.

As the relative humidity increases from 55% RH to 80% RH shown in Fig. 3a–c, the characteristic peak intensity of CH_3_NH_3_PbI_3_ is decreased, see the azimuth integration in the out-of-plane direction in Fig. 3d. With the rise of humidity, the peak intensity increase of 2D [PbI_6_]^4−^ octahedral pieces is not obvious due to the first degradation stage complete at the surface range, although the perovskite degradation continues in bulk. Compared with the network coverage of the CH_3_NH_3_PbI_3_ film on TiO_2_ nanoparticles, the surface morphology deteriorates significantly after the *in situ* humidity experiment (Fig. 3e). The crystal grains were partly separated and the porosity between the grains increased obviously. This indicates that new grain boundaries are generated accompanied by the breakage of perovskite crystalline structures, which is in accordance with the GIXRD analysis of CH_3_NH_3_PbI_3_ degradation in moisture.

**Fig. 4:**
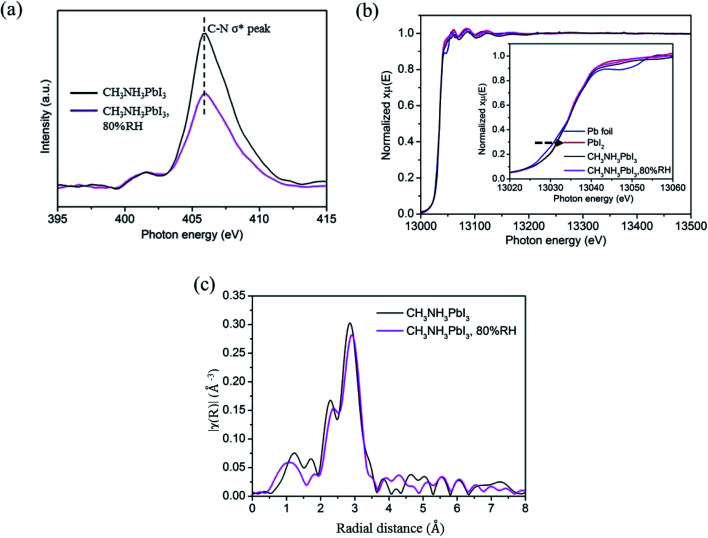
Schematic diagrams of XANES measurements of (a) nitrogen K-edge and (b) lead L-edge of CH_3_NH_3_PbI_3_ perovskite film before and after exposure to moisture. (c) EXAFS spectra in *R*-space of perovskite films before and after exposure to moisture with 80% relatively humidity.

An XANES study was performed to further examine the degradation of CH_3_NH_3_PbI_3_ on TiO_2_ nanoparticle films in moisture. The XANES measurement was carried out by using the total electron yield (TEY) mode under high vacuum conditions of 8 × 10^−6^ torr. The photon flux of the incident beam (*I*_0_) was simultaneously obtained by monitoring the photocurrent of the Au grid which is located in front of the entrance of the terminal station. In Fig. 4a, the K-edge XANES of element nitrogen probes N 2p electronic states, and the main peak at ∼406 eV is attributed to the C–N σ* transitions. The peak intensity decreases obviously after the CH_3_NH_3_PbI_3_/TiO_2_ NP films are exposed to moisture. It indicated that partial hydrogen bonds between organic amino and halide ions, as well as weak van der Waals interactions between organic components, were altered. The protons of the CH_3_NH_3_^+^ cation are extracted by the adhesive water molecules and decomposed into volatile methylamine, thereby reducing C–N σ* transitions.

In order to further investigate the influence of moisture, XAFS based on synchrotron radiation was used to detect the electronic structure of perovskite. The local environment of Pb was examined by measuring Pb L3-edge (Pb 2p_3/2_ core electron to *n*d unoccupied states) *via* fluorescence detection mode. The X-ray absorption near edge structure (XANES) and extended X-ray absorption fine structure (EXAFS) spectra were analyzed by standard procedure with Athena software package^49^ There is an obvious linear relationship between the absorption edge and the valence state, and the transition to high energy indicates that there is a higher valence state in the sample. Pb is commonly presented as a divalent cation (s^2^p^0^), the XANES spectra of all the samples are close to the Pb^2+^ state except the Pb^0^ state of standard Pb foil, as shown in Fig. 4b. The results showed that the chemical state of Pb did not change significantly in the *in situ* wet experiment, which indicates that the [PbI_6_]^4−^ octahedral crystals were not broken in the first stage of CH_3_NH_3_PbI_3_ degradation.

**Fig. 5:**
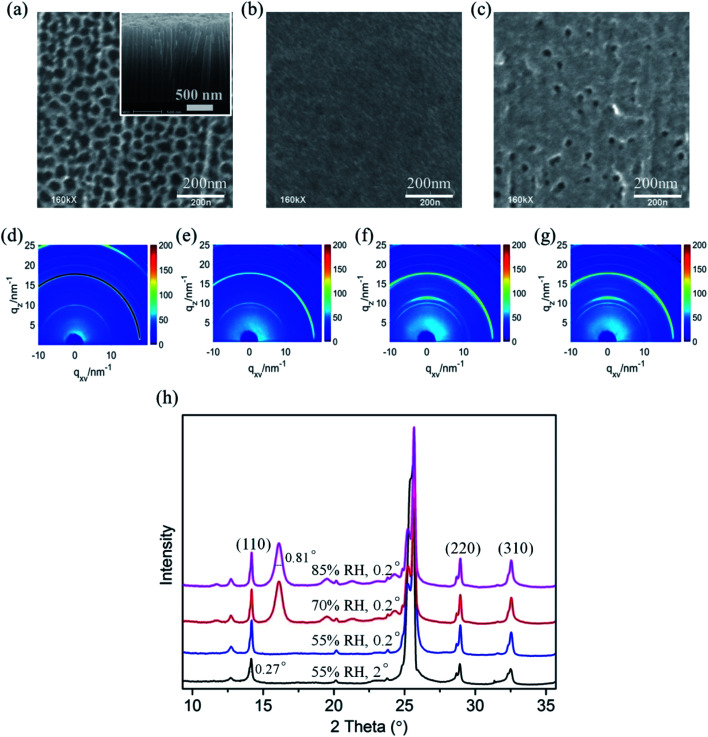
SEM images of (a) bare TiO_2_ nanotube films, (b) fabricated CH_3_NH_3_PbI_3_ films deposited on TiO_2_ nanotubes, and (c) degraded CH_3_NH_3_PbI_3_ films deposited on TiO_2_ nanotubes after the *in situ* humidity experiment. The inset of Fig. 2a is the cross-sectional SEM image of the vertical TiO_2_ nanotubes. (d and e) The 2D GIXRD patterns in *q* space of the CH_3_NH_3_PbI_3_/TiO_2_ films in the environment of 55% relative humidity, incident X-ray grazing angles of 2° and 0.2°, respectively. (f and g) The 2D GIXRD patterns of the CH_3_NH_3_PbI_3_/TiO_2_ films with relative humidity at 65% RH and 80% RH. (h) The exacted one-dimensional diffraction spectrum for Fig. 5d–g obtained by integrating the azimuth with the same *q*.

The tetragonal perovskite structure is not absolutely symmetrical, which means that the Pb–I bond length in the *b*-axis direction differs from that in the ac plane. The two peaks at 2–3 Å in *R*-space of EXAFS spectrum (Fig. 5c) could be specified as the two coordinating peaks of Pb–I, which are defined as Pb–I_1_ (*R* = 2.4 Å) and Pb–I_2_ (*R* = 2.8 Å).^50^ It can be observed that the two peaks move to large radial distance after exposure in 80% relative humidity, which suggests that the distance between the central Pb atom and the I atom is increased, that is, the Pb–I bond tends to unstable. The fitting results of Pb L3 EXAFS by scattering from a single coordination shell of iodine shown in Table S1† further prove the analyses. What's more, the Debye–Waller factor, namely the degree of disorder of CH_3_NH_3_PbI_3_ is significantly when it is exposed in 80% relative humidity, which provide evidence that the introduction of water will reduce crystal symmetry and increase the distortion of [PbI_6_]^4−^ octahedral, thereby generating some two-dimensional network of [PbI_6_]^4−^ octahedra intermediate and increasing the degree of disorder.

The comprehensive results obtained by *in situ* GIXRD and XANES measurements performed at controlled relative humidity demonstrate that intermediate [PbI_6_]^4−^ octahedral crystals are induced, which is strongly linked with the progress of chemical degradation in CH_3_NH_3_PbI_3_ perovskite films.

To confirm the intermediate crystal structure and evaluate the substrate effect on CH_3_NH_3_PbI_3_ stability in optoelectronics, crystalline anodic TiO_2_ nanotube arrays were introduced as substrates for perovskite CH_3_NH_3_PbI_3_ films. Highly ordered TiO_2_ nanotube arrays were fabricated on titanium foil by an electrochemical anodization method that we reported elsewhere.^51–53^ The morphologies of the bare TiO_2_ nanotubes with uniform regular pores can be observed in Fig. 5a, and the inset SEM image of the cross-sectional morphology confirms its vertical tubular structure. The tube arrays formed on the Ti foil are 2 μm in length and 60 nm in diameter, with a pore size of ∼50 nm. CH_3_NH_3_PbI_3_ perovskite film was prepared on TiO_2_ NT arrays by a sequential deposition method, which is described in the experiment section. The TiO_2_ nanotube arrays were perfectly covered by the CH_3_NH_3_PbI_3_ perovskite films with good uniformity, as shown in Fig. 5b.

Next, *in situ* GIXRD measurements were taken for the CH_3_NH_3_PbI_3_/TiO_2_ NTs to study their degradation in a moist environment. The 2D GIXRD pattern of such perovskite films under the environment of 50% RH is shown in Fig. 5d and e, with incident X-ray grazing angles of 2° and 0.2°, while Fig. 5f and g demonstrates their GIXRD patterns with higher relative humidity at 65% RH and 80% RH. The exacted one-dimensional diffraction spectrum for Fig. 5d–g is shown in Fig. 5h by integrating the azimuth with the same *q*. The three main crystal planes of (110), (220), and (310) for the tetragonal perovskite structure at the diffraction vectors *q* = 10, 20, and 22.1 nm^−1^ are the same as those of the CH_3_NH_3_PbI_3_/TiO_2_ NPs for all spectra. However, no split is observed for these crystal planes even at small grazing angles, which indicates that the crystal size of the CH_3_NH_3_PbI_3_ perovskite on the TiO_2_ nanotubes is uniform and smaller than that of the TiO_2_ nanoparticle films. The regular pores of the nanotubes likely confine the crystal growth and formation of the CH_3_NH_3_PbI_3_ perovskite.

No intermediate phase (2theta = 16°) is observed when the CH_3_NH_3_PbI_3_/TiO_2_ NTs are exposed to 50% RH moisture at both 2° and 0.2° grazing angles, which is different from the demonstrated CH_3_NH_3_PbI_3_/TiO_2_ NPs (Fig. 3d). When the controlled humidity is increased up to 65% RH, the same intermediate phase with an FWHM of 0.81° at *q* = 11 nm^−1^ (2theta = 16°) is observed. The crystallite sizes of the intermediate [PbI_6_]^4−^ octahedral crystals are calculated to be 7.9 nm by using the Scherrer equation, which is much smaller than that of the intermediate crystals in the degraded CH_3_NH_3_PbI_3_/TiO_2_ NPs. When the CH_3_NH_3_PbI_3_ perovskite is infiltrated into the surface of TiO_2_ nanotube arrays, the regular pores of such crystalline nanotubes probably confine the 3D [PbI_6_]^4−^ octahedral crystal network and restrain the intermediate formation induced by the broken bonds from organic amino cations. Thus, the degradation of the CH_3_NH_3_PbI_3_ perovskite using the TiO_2_ nanotube substrate is relatively slow compared with that of the conventional TiO_2_ nanoparticles in moisture.

The surface morphology of the CH_3_NH_3_PbI_3_/TiO_2_ NTs is shown in Fig. 5c after the *in situ* humidity experiment. Sporadic pores are observed compared with the uniform coverage of the CH_3_NH_3_PbI_3_ film (Fig. 5b). No cracks or new grain boundaries are found, which indicates that the deformation of perovskite crystals is restrained by the bottom TiO_2_ nanotube arrays, slowing the degradation of the CH_3_NH_3_PbI_3_/TiO_2_ NTs in a moist environment. The SEM observations are in accordance with the GIXRD analysis of the CH_3_NH_3_PbI_3_/TiO_2_ NT degradation in moisture.

**Fig. 6:**
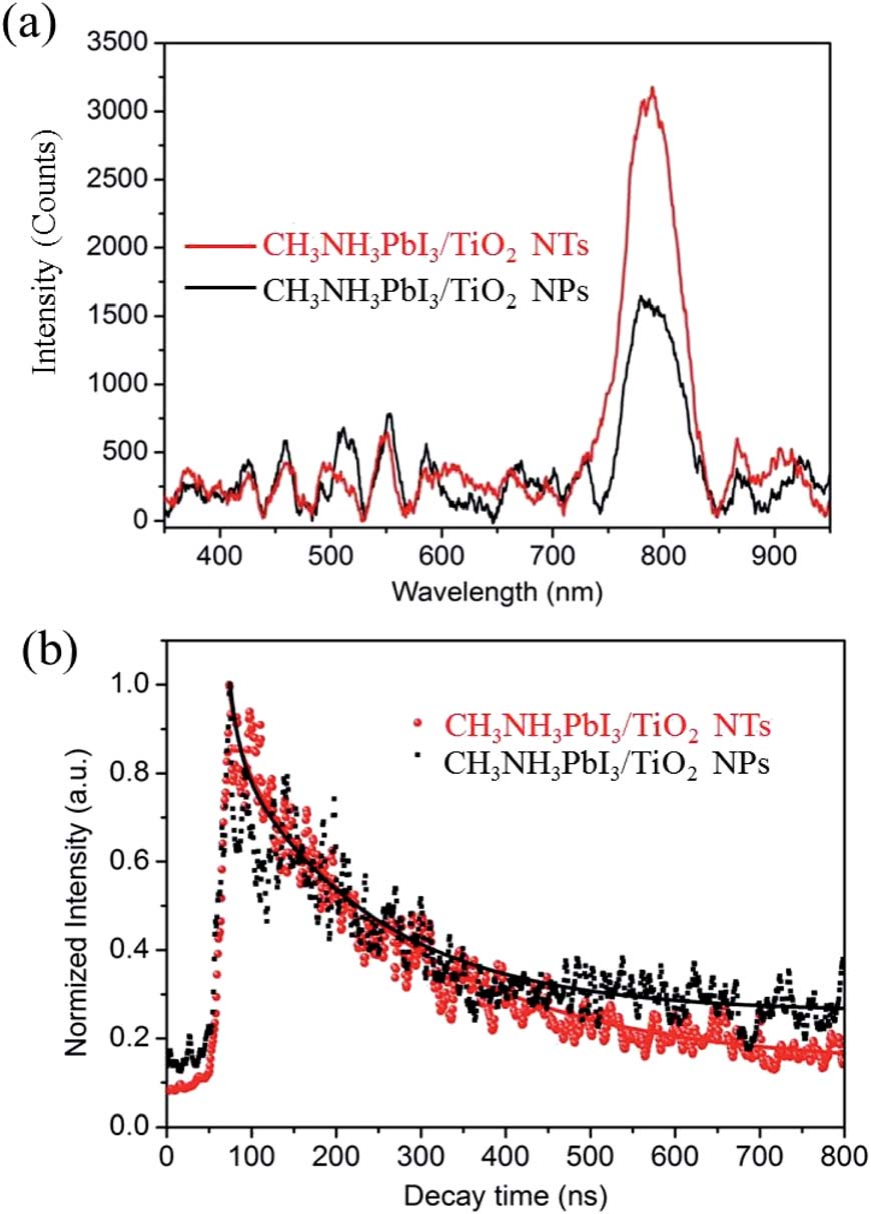
(a) XEOL spectra of CH_3_NH_3_PbI_3_/TiO_2_ NPs and CH_3_NH_3_PbI_3_/TiO_2_ NTs with 40 keV X-ray excitation. (b) The experimental data of the fluorescence decay curve at 790 nm excited with a 40 keV excitation, and the solid lines are fitting curves.

To evaluate the X-ray scintillation properties of the organometal halide perovskite film after incorporating the anodic TiO_2_ nanotube substrate, we measured the light output by XEOL for the as-prepared CH_3_NH_3_PbI_3_ films deposited on both TiO_2_ NP and TiO_2_ NT substrates with excitation at 40 keV. Fig. S4† shows the measurement setup for the XEOL emission spectrum. In this setup, a monochromatic X-ray beam illuminated the CH_3_NH_3_PbI_3_/TiO_2_ NTs at normal incidence, and the fluorescence emitted through the film was collected by an optical fiber and transferred to a grating spectrometer. The measured emission spectra of the CH_3_NH_3_PbI_3_/TiO_2_ NPs and CH_3_NH_3_PbI_3_/TiO_2_ NTs are shown in Fig. 6a. Both perovskite films demonstrate a single XEOL peak centered at 790 nm, which matches the band gaps (1.57 eV) of reported tetragonal CH_3_NH_3_PbI_3_ perovskites.^3,54,55^

The full width half maximums (FWHMs) of the XEOL peak are 57 nm and 53 nm for the CH_3_NH_3_PbI_3_/TiO_2_ NPs and CH_3_NH_3_PbI_3_/TiO_2_ NTs, respectively. The broad XEOL spectra are attributed to the high photon energy of the X-rays, in which the electrons are excited to high energy levels in the conduction band, resulting in a broad emission when the excited electrons relax to different lower energy levels. The enhancement of visible light output is observed between 750 nm and 850 nm for the CH_3_NH_3_PbI_3_ perovskite fabricated on TiO_2_ nanotube arrays. The XEOL peak intensity of the CH_3_NH_3_PbI_3_/TiO_2_ NTs is enhanced by 88% compared with that of the TiO_2_ nanoparticle films at 790 nm. Small organometal halide perovskites have a larger exciton binding energy than large crystals.^56^ Herein, the small crystal size of the CH_3_NH_3_PbI_3_ perovskite confined by TiO_2_ nanotubes contributes to the relatively high exciton binding energy, increasing exciton recombination and light output efficiency. In addition, the improved stability and crystallinity of the CH_3_NH_3_PbI_3_ crystals fabricated on TiO_2_ nanotubes contributes to its improved emission intensity.

The decay of fluorescence excited by the X-ray was measured using a Time-Correlated Single Photon Counting (TCSPC) method. Fluorescence decay profile can be fitted by a two exponential function, *I*(*t*) = *A*_0_ + *A*_1_ × exp(−*t*/*τ*_1_) + *A*_2_ × exp(−*t*/*τ*_2_), where *τ*_1_ and *τ*_2_ denote the decay times for the faster and the slower component, respectively, and *A*_1_ and *A*_2_ are the amplitudes, as presented in Table S2.† The dominant faster lifetimes *τ*_1_ for CH_3_NH_3_PbI_3_ prepared on both TiO_2_ nanotubes and TiO_2_ nanoparticles films at 790 nm are all about 12 ns, while the slower lifetimes *τ*_2_ are 220 and 170 ns at 790 nm, as shown in Fig. 5b. The faster and slower decay represent the non-radiative recombination and free exciton recombination, respectively. The lifetime for the CH_3_NH_3_PbI_3_ prepared on both TiO_2_ nanotubes are longer than that deposited on TiO_2_ nanoparticles, which demonstrates the perovskite CH_3_NH_3_PbI_3_ prepared on TiO_2_ nanotubes has good crystallinity and scintillation property with less crystal distortion.

Except for the CH_3_NH_3_PbI_3_ perovskite, the method of incorporating TiO_2_ nanotubes as a substrate is demonstrated to increase the scintillation properties in other systems. The light output for the CH_3_NH_3_PbBr_3_ perovskite prepared on TiO_2_ nanotubes is 75% higher than that on the TiO_2_ nanoparticle substrate, which has the same tendency as CH_3_NH_3_PbI_3_, see ESI Fig. S5.† Incorporating TiO_2_ nanotubes is likely a universal method to increase XEOL intensity and scintillation applications.

## 4. Conclusion

We used *in situ* high-resolution 2D GIXRD to determine the structural evolution of perovskite CH_3_NH_3_PbI_3_ prepared on both conventional porous TiO_2_ nanoparticles and anodic TiO_2_ nanotubes in controlled relative humidity. The GIXRD and XANES measurements suggest that some 2D network of [PbI_6_]^4−^ octahedra is constructed for the intermediate structure. We also show that the intermediate is observed once the CH_3_NH_3_PbI_3_/TiO_2_ NPs are exposed to humidity 50% RH, while the same intermediate appears at 65% RH for CH_3_NH_3_PbI_3_/TiO_2_ NTs. The regular pores of TiO_2_ nanotube arrays confine the 3D [PbI_6_]^4−^ octahedral network, reducing ion migration and octahedral agglomeration and improving the stability and crystallinity of the assisted organometal halide CH_3_NH_3_PbI_3_ perovskite. Compared with the conventional TiO_2_ nanoparticle substrate, the XEOL peak intensity of CH_3_NH_3_PbI_3_ is increased up to 88% by incorporating anodic TiO_2_ NTs. These results help elucidate fundamental decomposition pathways in organolead halide perovskite films and contribute paths for further scintillation applications in perovskites.

## Conflicts of interest

We declare that we have no conflict of interest.

## Supplementary Material

RA-010-D0RA08881A-s001

## References

[cit1] Qin P., Tanaka S., Ito S., Tetreault N., Manabe K., Nishino H., Nazeeruddin M. K., Grätzel M. (2014). Nat. Commun..

[cit2] Min H., Kim M., Lee S.-U., Kim H., Kim G., Choi K., Lee J. H., Seok S. I. (2019). Science.

[cit3] Kim H.-S., Lee C.-R., Im J.-H., Lee K.-B., Moehl T., Marchioro A., Moon S.-J., Humphry-Baker R., Yum J.-H., Moser J. E., Grätzel M., Park N.-G. (2012). Sci. Rep..

[cit4] Xing G., Mathews N., Sun S., Lim S. S., Lam Y. M., Grätzel M., Mhaisalkar S., Sum T. C. (2013). Science.

[cit5] Gedamu D., Asuo I. M., Benetti D., Basti M., Ka I., Cloutier S. G., Rosei F., Nechache R. (2018). Sci. Rep..

[cit6] Li X., Bi D., Yi C., Décoppet J.-D., Luo J., Zakeeruddin S. M., Hagfeldt A., Grätzel M. (2016). Science.

[cit7] Ponseca C. S., Savenije T. J., Abdellah M., Zheng K., Yartsev A., Pascher T., Harlang T., Chabera P., Pullerits T., Stepanov A., Wolf J.-P., Sundström V. (2014). J. Am. Chem. Soc..

[cit8] Zhou H., Chen Q., Li G., Luo S., Song T.-B., Duan H.-S., Hong Z., You J., Liu Y., Yang Y. (2014). Science.

[cit9] Chen W., Wu Y., Yue Y., Liu J., Zhang W., Yang X., Chen H., Bi E., Ashraful I., Grätzel M., Han L. (2015). Science.

[cit10] Yang W. S., Park B.-W., Jung E. H., Jeon N. J., Kim Y. C., Lee D. U., Shin S. S., Seo J., Kim E. K., Noh J. H., Seok S. I. (2017). Science.

[cit11] Sahli F., Werner J., Kamino B. A., Bräuninger M., Monnard R., Paviet-Salomon B., Barraud L., Ding L., Diaz Leon J. J., Sacchetto D., Cattaneo G., Despeisse M., Boccard M., Nicolay S., Jeangros Q., Niesen B., Ballif C. (2018). Nat. Mater..

[cit12] Wang L., Zhou H., Hu J., Huang B., Sun M., Dong B., Zheng G., Huang Y., Chen Y., Li L., Xu Z., Li N., Liu Z., Chen Q., Sun L.-D., Yan C.-H. (2019). Science.

[cit13] Jung E.
H., Jeon N. J., Park E. Y., Moon C. S., Shin T. J., Yang T.-Y., Noh J. H., Seo J. (2019). Nature.

[cit14] Lu H., Liu Y., Ahlawat P., Mishra A., Tress W. R., Eickemeyer F. T., Yang Y., Fu F., Wang Z., Avalos C. E., Carlsen B. I., Agarwalla A., Zhang X., Li X., Zhan Y., Zakeeruddin S. M., Emsley L., Rothlisberger U., Zheng L., Hagfeldt A., Grätzel M. (2020). Science.

[cit15] Zhao B., Bai S., Kim V., Lamboll R., Shivanna R., Auras F., Richter J. M., Yang L., Dai L., Alsari M., She X.-J., Liang L., Zhang J., Lilliu S., Gao P., Snaith H. J., Wang J., Greenham N. C., Friend R. H., Di D. (2018). Nat. Photonics.

[cit16] Tan Z.-K., Moghaddam R. S., Lai M. L., Docampo P., Higler R., Deschler F., Price M., Sadhanala A., Pazos L. M., Credgington D., Hanusch F., Bein T., Snaith H. J., Friend R. H. (2014). Nat. Nanotechnol..

[cit17] Xing J., Yan F., Zhao Y., Chen S., Yu H., Zhang Q., Zeng R., Demir H. V., Sun X., Huan A., Xiong Q. (2016). ACS Nano.

[cit18] Chin X. Y., Cortecchia D., Yin J., Bruno A., Soci C. (2015). Nat. Commun..

[cit19] Perumal A., Shendre S., Li M., Tay Y. K. E., Sharma V. K., Chen S., Wei Z., Liu Q., Gao Y., Buenconsejo P. J. S., Tan S. T., Gan C. L., Xiong Q., Sum T. C., Demir H. V. (2016). Sci. Rep..

[cit20] Wong A. B., Lai M., Eaton S. W., Yu Y., Lin E., Dou L., Fu A., Yang P. (2015). Nano Lett..

[cit21] Palma A. L., Cinà L., Busby Y., Marsella A., Agresti A., Pescetelli S., Pireaux J.-J., Di Carlo A. (2016). ACS Appl. Mater. Interfaces.

[cit22] Huang C., Zhang C., Xiao S., Wang Y., Fan Y., Liu Y., Zhang N., Qu G., Ji H., Han J., Ge L., Kivshar Y., Song Q. (2020). Science.

[cit23] Qin C., Sandanayaka A. S. D., Zhao C., Matsushima T., Zhang D., Fujihara T., Adachi C. (2020). Nature.

[cit24] Yakunin S., Sytnyk M., Kriegner D., Shrestha S., Richter M., Matt G. J., Azimi H., Brabec C. J., Stangl J., Kovalenko M. V., Heiss W. (2015). Nat. Photonics.

[cit25] Shrestha S., Fischer R., Matt G. J., Feldner P., Michel T., Osvet A., Levchuk I., Merle B., Golkar S., Chen H., Tedde S. F., Schmidt O., Hock R., Rührig M., Göken M., Heiss W., Anton G., Brabec C. J. (2017). Nat. Photonics.

[cit26] Kim Y. C., Kim K. H., Son D.-Y., Jeong D.-N., Seo J.-Y., Choi Y. S., Han I. T., Lee S. Y., Park N.-G. (2017). Nature.

[cit27] Steele J. A., Pan W., Martin C., Keshavarz M., Debroye E., Yuan H., Banerjee S., Fron E., Jonckheere D., Kim C. W., Baekelant W., Niu G., Tang J., Vanacken J., Van Der Auweraer M., Hofkens J., Roeffaers M. B. J. (2018). Adv. Mater..

[cit28] Ye F., Lin H., Wu H., Zhu L., Huang Z., Ouyang D., Niu G., Choy W. C. H. (2019). Adv. Funct. Mater..

[cit29] Náfrádi B., Náfrádi G., Forró L., Horváth E. (2015). J. Phys. Chem. C.

[cit30] Zhang Y., Sun R., Ou X., Fu K., Chen Q., Ding Y., Xu L.-J., Liu L., Han Y., Malko A. V., Liu X., Yang H., Bakr O. M., Liu H., Mohammed O. F. (2019). ACS Nano.

[cit31] Grancini G., Roldán-Carmona C., Zimmermann I., Mosconi E., Lee X., Martineau D., Narbey S., Oswald F., De Angelis F., Graetzel M., Nazeeruddin M. K. (2017). Nat. Commun..

[cit32] Correa-Baena J.-P., Saliba M., Buonassisi T., Grätzel M., Abate A., Tress W., Hagfeldt A. (2017). Science.

[cit33] Christians J. A., Miranda Herrera P. A., Kamat P. V. (2015). J. Am. Chem. Soc..

[cit34] Noh J. H., Im S. H., Heo J. H., Mandal T. N., Seok S. I. (2013). Nano Lett..

[cit35] Tian Y., Zhou C., Worku M., Wang X., Ling Y., Gao H., Zhou Y., Miao Y., Guan J., Ma B. (2018). Adv. Mater..

[cit36] Niu G., Guo X., Wang L. (2015). J. Mater. Chem. A.

[cit37] Yang J., Siempelkamp B. D., Liu D., Kelly T. L. (2015). ACS Nano.

[cit38] Hu Q., Zhao L., Wu J., Gao K., Luo D., Jiang Y., Zhang Z., Zhu C., Schaible E., Hexemer A., Wang C., Liu Y., Zhang W., Grätzel M., Liu F., Russell T. P., Zhu R., Gong Q. (2017). Nat. Commun..

[cit39] Frost J. M., Butler K. T., Brivio F., Hendon C. H., Van Schilfgaarde M., Walsh A. (2014). Nano Lett..

[cit40] Etgar L., Gao P., Xue Z., Peng Q., Chandiran A. K., Liu B., Nazeeruddin M. K., Grätzel M. (2012). J. Am. Chem. Soc..

[cit41] Baikie T., Fang Y., Kadro J. M., Schreyer M., Wei F., Mhaisalkar S. G., Graetzel M., White T. J. (2013). J. Mater. Chem. A.

[cit42] Wang Z.-K., Li M., Yang Y.-G., Hu Y., Ma H., Gao X.-Y., Liao L.-S. (2016). Adv. Mater..

[cit43] Maryam S., Saidah R., Mufti N., Sunaryono S., Fuad A. (2019). Mater. Today: Proc..

[cit44] Kawamura Y., Mashiyama H., Hasebe K. (2002). J. Phys. Soc. Jpn..

[cit45] Peng W., Yin J., Ho K.-T., Ouellette O., De Bastiani M., Murali B., El Tall O., Shen C., Miao X., Pan J., Alarousu E., He J.-H., Ooi B. S., Mohammed O. F., Sargent E., Bakr O. M. (2017). Nano Lett..

[cit46] Hirasawa M., Ishihara T., Goto T. (1994). J. Phys. Soc. Jpn..

[cit47] Ghodke S., Sonawane S., Gaikawad R., Mohite K. C. (2012). Can. J. Chem. Eng..

[cit48] Hernández I., Maubert A., Rendon Vazquez L., Santiago P., Hernández H., Arceo L., Febles V., González E. P., González-Reyes L. (2012). Int. J. Electrochem. Sci..

[cit49] Liu B., Cui R., Huang H., Guo X., Zuo S., Dong J., Yao H., Li Y., Zhao D., Wang J., Zhang J., Chen Y., Yang J., Sun B. (2020). Sol. Energy.

[cit50] Gilbert B., Frazer B. H., Belz A., Conrad P. G., Nealson K. H., Haskel D., Lang J. C., Srajer G., De Stasio G. (2003). J. Phys. Chem. A.

[cit51] Li H., Chen Z., Tsang C. K., Li Z., Ran X., Lee C., Nie B., Zheng L., Hung T., Lu J., Pan B., Li Y. Y. (2014). J. Mater. Chem. A.

[cit52] Li H., Zhang J., Cheng J.-W., Chen Z., Liang F., Tsang C.-K., Cheng H., Zheng L., Lee S.-T., Li Y. (2012). ECS J. Solid State Sci. Technol..

[cit53] Li H., Cheng J.-W., Shu S., Zhang J., Zheng L., Tsang C. K., Cheng H., Liang F., Lee S.-T., Li Y. Y. (2013). Small.

[cit54] Lee M. M., Teuscher J., Miyasaka T., Murakami T. N., Snaith H. J. (2012). Science.

[cit55] Kojima A., Teshima K., Shirai Y., Miyasaka T. (2009). J. Am. Chem. Soc..

[cit56] Kumar S., Jagielski J., Yakunin S., Rice P., Chiu Y.-C., Wang M., Nedelcu G., Kim Y., Lin S., Santos E. J. G., Kovalenko M. V., Shih C.-J. (2016). ACS Nano.

