# Supplementary material for: Stability study of organometal halide perovskite and its enhanced X-ray scintillation from the incorporation of anodic TiO2 nanotubes
Source: RSC Adv. 2020 Dec 8;10(71):43773–82. doi: 10.1039/d0ra08881a (PMC9058416; doi:10.1039/d0ra08881a)
Supplement: RA-010-D0RA08881A-s001 [file RA-010-D0RA08881A-s001.pdf]

Electronic Supplementary Material (ESI) for RSC Advances.
This journal is © The Royal Society of Chemistry 2020

Stability study of halide perovskite and its enhanced X-ray scintillation from the incorporating of anodic TiO_2 nanotubes

Hui Li,[a] Zhenhua Chen,[b]* Zhuocheng Sang,[a] Xiangzhi Zhang,[b] Yong wang [b]

[a]Department of Applied Physics, Donghua University, Songjiang District, Shanghai 201620, China
[b]Shanghai Synchrotron Radiation Facility (SSRF), Shanghai Advanced Research Institute, Chinese Academy of Sciences, Shanghai 201800, China

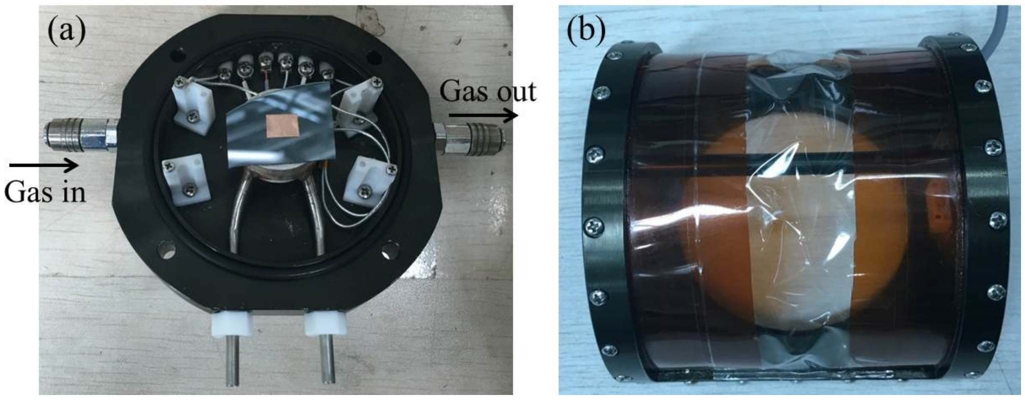

Figure S1. Schematic of the spectrometric measurement setup mounted on the BL14B beamline.

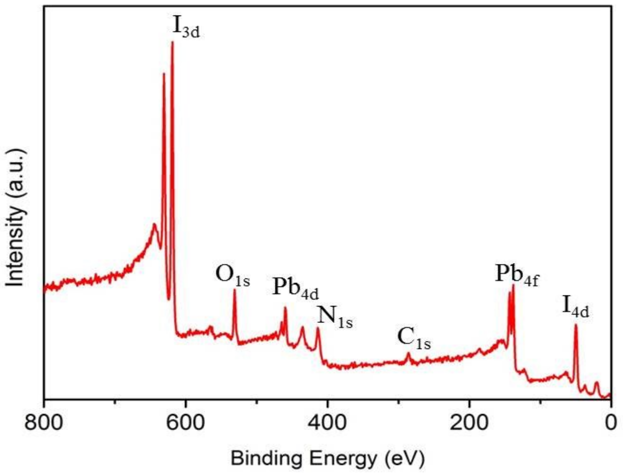

Figure S2. XPS measurements for perovskite $CH_3NH_3PbI_3$ film deposited on FTO glass.

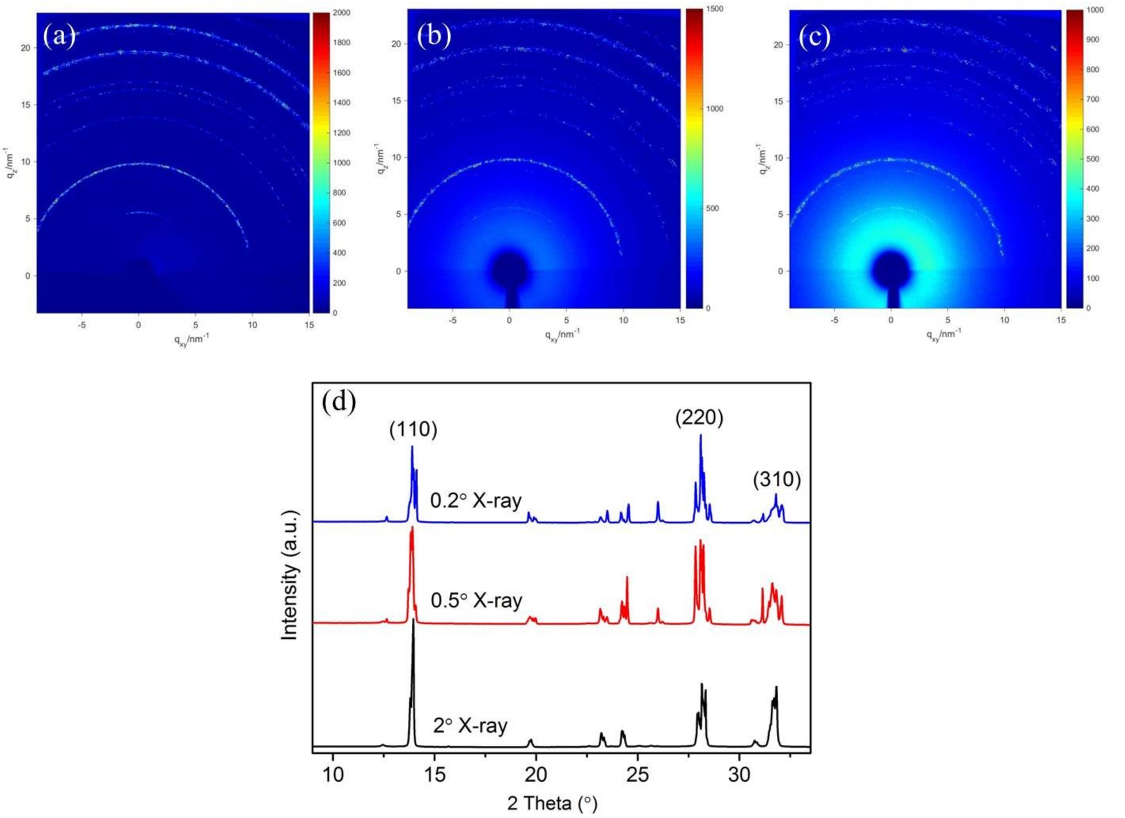

Figure S3. (a)-(c) The two-dimensional diffraction signals of the as prepared $CH_3NH_3PbI_3/TiO_2$ NP films using incident measurement grazing angles of 2°, 0.5°, and 0.2°. (d) The dimensional diffraction spectrum integrated from Figure S3a-3c in the out-of-plane direction.

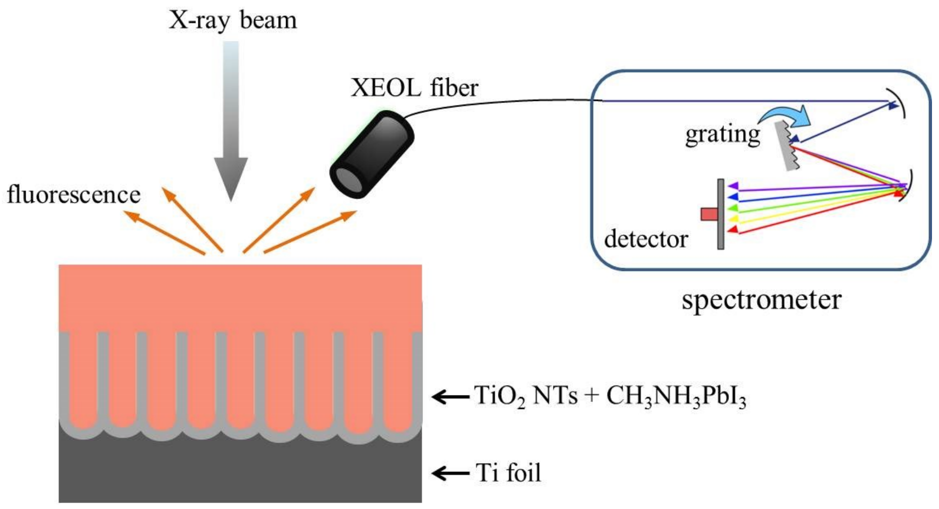

Figure S4. Schematic of the spectrometric measurement setup mounted on the BL13W beamline at SSRF.

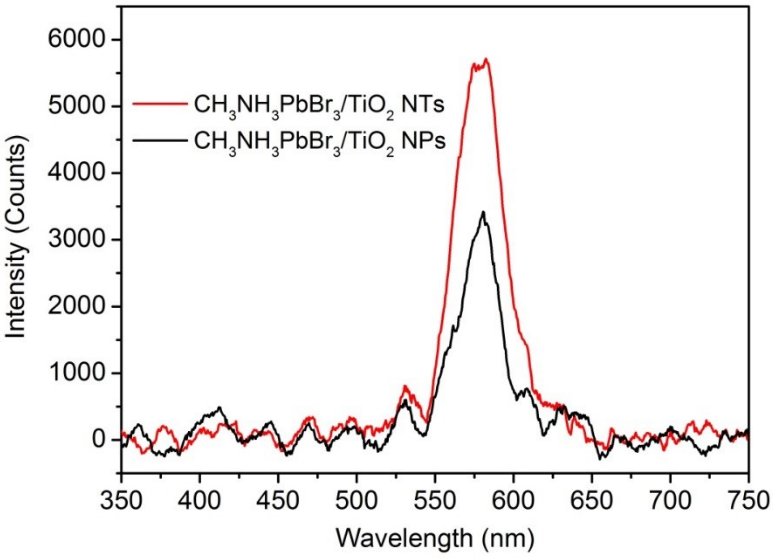

Figure S5. XEOL spectra of $CH_3NH_3PbBr_3/TiO_2$ NPs and $CH_3NH_3PbBr_3/TiO_2$ NTs with 40 keV X-ray excitation.

Table S1. Parameters obtained from fitting the Pb L3 EXAFS by scattering from a single coordination shell of iodine.

Sample	Atomic number, N^a	Amplitude damping factor, S_0^2	Debye-Waller factor, σ^2 (Å^2)	Bond length, Pb-I_2, r (Å)	R factor
$CH_3NH_3PbI_3$	6	0.6777	0.0165	3.1415	0.0089
$CH_3NH_3PbI_3$, 80%RH	6	0.7037	0.0176	3.1858	0.0037

Table S2. Fitted parameters for $CH_3NH_3PbI_3$ samples prepared on TiO_2 nanotubes or TiO_2 nanoparticles.

Fluorescence decay profiles were fitted by exponential function equation: $I(t)=A_0+A_1*exp(^{-}t/\tau_1)+A_2*exp(^{-}t/\tau_2)$. τ_1 and τ_2 denote the decay time for the faster and the slower components, and A_1 and A_2 are the fluorescence amplitudes.

Sample	A_0	τ_1 (ns)	τ_2 (ns)	A_1	A_2
$CH_3NH_3PbI_3/TiO_2$ nanotubes at (790 nm)	0.14	12.9	220	40	1
$CH_3NH_3PbI_3/TiO_2$ nanoparticles at (790 nm)	0.26	12	170	80	0.9
